# Adaptation of an evidence-based postpartum depression intervention: feasibility and acceptability of mothers and babies 1-on-1

**DOI:** 10.1186/s12884-018-1726-0

**Published:** 2018-04-11

**Authors:** S. Darius Tandon, Julie A. Leis, Erin A. Ward, Hannah Snyder, Tamar Mendelson, Deborah F. Perry, Mya Carter, Jaime Hamil, Huynh-Nhu Le

**Affiliations:** 1 Northwestern University Feinberg School of Medicine, 750 N. Lake Shore Drive, 6th Floor, Chicago, IL 60614 USA; 2 James Bell Associates, 3033 Wilson Blvd, Suite 650, Arlington, VA 22201 USA; 3 Department of Psychology, George Washington University, 2125 G. St. NW, Washington, DC 20052 USA; 4 Department of Mental Health, Johns Hopkins University Bloomberg School of Public Health, 624 N. Broadway, Room 853, Baltimore, MD 21205 USA; 5 Center for Child and Human Development, Georgetown University, 3300 Whitehaven St., Suite 3300, Box 571485, Washington, DC 20057 USA

**Keywords:** Home visiting, Depression, Intervention, Prevention, Implementation

## Abstract

**Background:**

Mothers and Babies (MB) is a cognitive-behavioral intervention with demonstrated efficacy in reducing depressive symptoms and preventing depressive episodes among perinatal women when delivered in a group format by mental health professionals. Study aims were to describe the adaptation of MB into a 1-on-1 modality (MB 1-on-1) and provide data on the adapted intervention’s acceptability and feasibility.

**Methods:**

Seventy-five home visitors trained on MB 1-on-1 delivered the 15-session intervention to 1–2 clients. Client acceptability data assessed intervention enjoyment, comprehension, and usefulness. Home visitor feasibility and acceptability data measured amount of intervention material delivered, client comprehension, and client engagement.

**Results:**

Home visitors were all female with 8.8 years of experience on average. 117 clients completed acceptability surveys. Average client age was 21.9 years and 41% were pregnant. Home visitors completely covered 87.9% of sessions and reported clients totally understood MB material 82.5% of the time across sessions, although variability was found in comprehension across modules. 82.0% of clients found MB 1-on-1 enjoyable and 91.6% said they totally understood sessions, when averaged across sessions. Clients enjoyed content on noticing one’s mood and pleasant activities. Implementation challenges were client engagement, facilitating completion of personal projects, and difficulty shifting between didactic and interactive activities.

**Conclusions:**

Clients found MB 1-on-1 to be enjoyable, easily understood, and useful. Home visitors reported excellent implementation fidelity and felt clients understood MB material. A refined 12-session version of MB 1-on-1 should be examined for its effectiveness in reducing depressive symptoms, given encouraging feasibility and acceptability data.

**Electronic supplementary material:**

The online version of this article (10.1186/s12884-018-1726-0) contains supplementary material, which is available to authorized users.

## Background

The prevalence of postpartum depression has been estimated at 10–22% and disproportionately affects low-income women of all racial and ethnic groups, with a 20% prevalence rate consistently found among low-income women [1–4]. Postpartum depression is a well-established risk factor for poor parenting practices, with strong and consistent evidence indicating that women experiencing postpartum depression exhibit less maternal responsiveness or sensitivity, less verbal and visual interaction, and more intrusiveness [5–8]. Disparities also exist across socioeconomic lines with respect to prevalence of clinically relevant perinatal depressive symptoms that do not meet DSM criteria for major depression. Studies indicate that rates of clinically elevated perinatal depressive symptoms among low-income women are 30–45% [9, 10] —double the rates of women of higher socio-economic status; these disparities exist irrespective of race, ethnicity, or geography [11–13]. Notably, the presence of subthreshold depressive symptoms are associated with many of the same negative maternal and child health outcomes as major depression [14, 15].

Systematic reviews have highlighted an array of efficacious interventions that reduce the risk of developing postpartum depression [16]. Among interventions that have demonstrated efficacy, the majority use health (e.g., nurses, midwives) or mental health (e.g., psychologists) professionals to deliver individualized or group-based interventions [16]. One exception is the use of peers to deliver support via phone [17], although this study was conducted in Canada with predominately White, upper and middle class women. To our knowledge, there are no interventions led by *non-health or non-mental health professionals* that have demonstrated efficacy in preventing the onset of postpartum depression and reduction of depressive symptoms among low-income women. Interventions delivered by non-health or non-mental health professionals have considerable potential to address the mental health needs of perinatal women, given the stigma associated with seeking mental health services, lack of knowledge about, or limited access to available mental health services [18, 19].

Home visiting programs (HV) are one setting through which perinatal women can receive mental health services via paraprofessionals. Current estimates indicate that more than 750,000 women receive HV services each year, making HV one of the largest avenues through which perinatal women come to the attention of service providers [20]. Home visiting is a service delivery strategy wherein trained paraprofessional or professionals (e.g., social workers, nurses) meet regularly in-home with pregnant women and women with young children. Twenty evidence-based HV models have been identified [20], which—depending on the model—focus on child development and school readiness, positive parenting practices, reduction in child maltreatment, improvement of birth outcomes, and promotion of maternal health. Across different HV models, home visitors tend to see clients weekly or biweekly. Core content of evidence-based HV models typically address: (a) preparation for childbirth and parenting, (b) provision of emotional and tangible support, (c) discussion of infant and child development, (d) linkages to prenatal and pediatric care, and (e) referrals for social and health services.

There is evidence that depression is challenging for HV programs to address and can negatively impact the effects of HV programs on their desired maternal and child health outcomes. Depressed clients in HV require more effort to engage in services [21] and home visitors often feel they do not have appropriate training on how to discuss depression with their clients [22]. Depressive symptoms among HV clients were associated with less parenting self-confidence, higher risk beliefes about parenting and knowledge of child development, and smaller social support networks [23] with other studies indicating that depressed HV clients exhibit more dysfunctional parent-child interactions [24] Recent years have seen increasing calls for and integration of depression screening into HV programs, including through the federal Maternal, Infant, and Early Childhood Home Visiting Program (MIECHV) initiative [25]. Although screening for depression is an important step, research indicates that HV clients who screen positive rarely receive community mental health services [26, 27]. As such, there is a growing need to identify strategies for providing services to HV clients to address this population’s mental health needs, which, in turn, may promote greater HV effectiveness in achieving its core outcomes.

To address maternal depression in home visiting, our study team collaborated with HV programs to integrate and evaluate the Mothers and Babies (MB) Course’s efficacy in preventing the onset of postpartum depression and worsening of depressive symptoms among HV clients. The MB Course is a manualized cognitive-behavioral intervention that promotes healthy mood management by teaching individuals ways to increase the frequency of thoughts and behaviors that lead to positive mood states. There are three MB modules: (1) behavioral activation to increase enjoyable activities, (2) restructuring harmful cognitions, and (3) increasing social support. MB content is tailored to specific needs and issues related to the perinatal period. A series of randomized controlled trials has demonstrated the association of MB with prevention of major depression onset, reduced depressive symptoms, and improved mood management among perinatal women receiving MB compared to women receiving usual care when MB is delivered in a group format by mental health professionals [28–32]. The most recent of these RCT’s have been conducted in HV settings [29, 31, 32].

Despite results showing MB is efficacious in addressing postpartum depression, there are limitations to mental health professionals delivering groups in HV settings: insufficient availability of clinicians, especially in rural areas, untenable costs associated with hiring clinicians, as well as barriers to client attendance such as transportation issues, childcare, and the time commitment to attend group sessions [33]. In response to these challenges, our research team developed and piloted a 1-on-1 adaptation of MB (MB 1-on-1). This article describes the MB 1-on-1 intervention, providing client and home visitor data on the feasibility and acceptability of implementation.

## Method

### Development of MB 1-on-1

The goal of developing MB 1-on-1 was to adapt intervention content to complement the educational information and supportive communications home visitors provide during visits into 15–20 min segments that could be effectively incorporated during regular home visits. In doing so, care was taken to retain core content of the MB Group curriculum while modifying the intervention to ensure its appropriateness for 1-on-1 delivery. As a starting point, all group activities requiring small or large group conversations were identified; those that required multiple group members were either revised for provider-client interactions or removed. Weekly group relaxation activities were also removed due to the time requirement, while a session teaching relaxation techniques was included in MB 1-on-1. We then removed material that was duplicative across sessions (e.g., multiple discussions of harmful vs. helpful thoughts in the Thoughts module became the focus of a single MB 1-on-1 session). This process resulted in a 15-session version of MB 1-on-1.

Along with modifying the content to be delivered in 15 brief sessions, considerable effort was placed on revising the Instructor’s Manual to make it user-friendly for home visitors accessing the Manual in a client’s home. Each session consists of two to three topics and a personal project (i.e., homework) for completion between sessions. All topics contain: a) key points for home visitors to communicate to clients as takeaway messages, b) a script to guide home visitors when delivering content, and c) interactive learning activities that help clients practice and understand key concepts.

### Setting

In partnership with the Illinois Governor’s Office for Early Childhood Development (OECD) an open invitation was offered to HV programs across Illinois to attend one of three regional day and a half-long trainings led by the first author on MB 1-on-1 between February and August 2014. Eighty-one home visitors from 19 HV programs in Illinois attended one of the trainings. Across programs, 14 (74%) used the Healthy Families America model, and 5 (26%) programs employed the Parents as Teachers model. The number of home visitors employed by agency ranged from 1 to 7. Healthy Families America enrolment targets parents facing challenges such as single parenthood, low income, childhood history of abuse and other adverse child experiences, and current or previous issues related to substance abuse, mental health issues, or domestic violence. Individual Healthy Families’ sites are given latitude to select specific characteristics of the target population they plan to serve, while enrolling prenatally or within three months of birth [34]. Parents as Teachers provider organizations select the specific characteristics and eligibility criteria of the target population they plan to serve, such as children with special needs, families at risk for child abuse, income-based criteria, teen parents, first-time parents, immigrant families, low literate families, or parents with mental health or substance use issues, enrolling families during pregnancy and the postpartum period [35].

During training, home visitors were provided with an orientation to MB’s cognitive-behavioral therapy underpinnings followed by an orientation to implementation guidelines and structure of the Instructor and Participant Manuals. The remainder of the training guided home visitors through each of the 15 sessions. Didactic portions of the training introduced home visitors to key points within each session and potential challenges to communicating material with clients. Training provided ample time for attendees to engage in small and large group activities, including opportunities to practice introducing core MB content, provider-client interactive activities, and personal project presentation, through structured role plays.

OECD was interested in training its HV programs on how to better address maternal depression among its clients and encouraged all HV programs trained to immediately pilot the intervention. Specifically, home visitors attending a training were asked to to implement MB with 1 to 2 clients who were pregnant or had a child < 6 months. In cases where prenatal and recently postpartum clients were not available, home visitors were encouraged to implement MB with clients on their caseload who could benefit from the intervention, with the goal being to practice implementation in close proximity to the training and supervision offered.

### Intervention implementation

MB 1-on-1 recommends session delivery every 1–2 weeks, with flexibility to conduct more than one session per week if a home visitor believes the material is particularly salient for a client. Home visitors were trained to deliver a MB session at the beginning or end of a scheduled home visit, with flexibility to conduct a MB session by phone if a client was unable to complete the scheduled home visit. Other individuals were permitted to be present during MB delivery (e.g., father of baby); however, home visitors were encouraged to use professional judgment regarding whether the presence of another individual would compromise MB delivery or client openness. HV clients referred to material in the Participant Manual during sessions. Personal projects for client completion between sessions were described in the Participant Manual. Home visitors were trained to implement MB 1-on-1 chronologically from session 1 through 15.

Each HV program received biweekly phone supervision from the first author upon beginning MB implementation. Supervision was provided for 4–6 months, which corresponded with the length of time it took, on average, for home visitors at a HV program to implement the 15-session curriculum. Supervision calls allowed home visitors to share and receive feedback on both content and implementation process. For example, home visitors posed content-related questions on how to help clients better understand a core MB concept or process-related questions on how to most effectively transition from delivering core HV content to MB material.

### Data sources and data collection

The Northwestern University Institutional Review Board reviewed and approved the study protocol and all data collection activities described herein. This protocol was deemed as minimal risk and pursuant to quality assurance activities and was therefore granted a waiver of consent.

#### Client acceptability

At the end of each of the 15 MB sessions, clients were asked to complete a brief de-identified 5-question survey given to them by their home visitor. Each survey asked the same questions: a) how much did you enjoy today’s session (1 = not enjoyable at all, 2 = somewhat enjoyable, 3 = very enjoyable), b) how well did you understand the information in today’s session (1 = didn’t understand, 2 = somewhat understood, 3 = totally understood), and c) how useful was the information in today’s session (1 = not useful at all, 2 = somewhat useful, 3 = very useful). Two open-ended questions were asked: do you have any suggestions on how to make today’s session any better, and, was there anything you really enjoyed or thought was really useful in today’s session.

#### Home visitor feasibility & acceptability

Home visitors also completed a survey at the end of each session. Each survey asked the home visitor to indicate for each session topic: a) how much material was covered (not covered, somewhat covered, totally covered), b) how well you think the client understood the topic (did not understand at all, somewhat understood, totally understood), and c) how engaged the client was (not engaged at all, somewhat engaged, very engaged). Home visitors were also given the opportunity to provide open-ended responses indicating if there were challenges when discussing session topics.

### Data analysis

Home visitors entered their data directly into a web-based Research Electronic Data Capture (REDCap) survey or emailed or faxed data to the research team who entered the data into REDCap. Client data were collected by home visitors and emailed or faxed to the research team for REDCap data entry. All data were subsequently exported to SPSS 22 for analysis. To analyze client acceptability data, we assessed *enjoyment* by calculating the percentage of women who indicated they found each session very enjoyable, somewhat enjoyable, or not enjoyable at all. We assessed content *comprehension* by calculating the percentage of women who indicated they didn’t understand, somewhat understood, or totally understood each session. We assessed perceived usefulness of MB skills by calculating the percentage of women who indicated they found skills not useful at all, somewhat useful, or very useful.

To assess home visitor feasibility and acceptability data, we examined *fidelity of MB implementation* by calculating the percentage of home visitors who reported that they totally covered, somewhat covered, or did not cover each topic within a session. Each session contains between 2 and 4 topics; the percentages for each topic were combined into an average score indicating the extent to which a session was covered. We assessed *client comprehension* by calculating the percentage of home visitors who indicated that a client totally understood, somewhat understood, or did not understand at all the session material for each session. We also estimated *client engagement* by calculating the percentage of home visitors who indicated that a client was totally engaged, somewhat engaged, or not engaged at all for each session.

For each client and home visitor acceptability and feasibility construct, we calculated percentages: a) across all 15 sessions (i.e., the entire intervention) and b) for each MB module [Sessions 1–3 (Overview), Sessions 4–6 (Pleasant Activities); Sessions 7–11 (Thoughts), Sessions 12–15 (Contacts with Others)]. All open-ended client and home visitor responses were exported into Microsoft Excel and coded inductively by a member of the research team; open-ended responses were provided by 95% of clients and 91% of home visitors. The same research team member created two matrices—one for client data and another for home visitor data. Each matrix had a row for each code and a column for each respondent and all open-ended data were placed in one of these matrices. These matrices were then used to guide analysis that identified the most common themes and patterns [36] related to the constructs that the open-ended questions asked about—for example, client enjoyment and home visitors perceptions of intervention fidelity.

## Results

### Home visitor demographics

Of the 81 home visitors trained, 75 implemented MB 1-on-1. Reasons that home visitors did not implement the intervention included: one HV program decided not to implement MB due to organizational transitions shortly after training (*n* = 3) and staff leaving the agency shortly after training (n = 3). Of the 75 who did implement MB 1-on-1, 68 provided data on implementation. The average age of these 68 female home visitors was 42 years (range 22–66) and the average length of time working as a home visitor was 8.8 years (range < 1 year - 24 years). The majority had at least a 2-year Associate’s Degree (72%). The race/ethnicity composition was 36% African American, 34% White, 25% Latina, 3% Asian-American, and 2% biracial.

### Client characteristics

A total of 129 clients received the MB 1-on-1 intervention delivered by the 75 home visitors, of whom 117 (91%) completed client acceptability surveys. Among the 117 study participants, the average age was 21.9 (range 15–36). Forty-five percent of women were African American, 28% White, and 27% Latina. A slight majority of clients (59%) had already delivered their baby when they started receiving MB, with the average number of weeks postpartum being 20 (range 1–96). The remaining 41% of women were pregnant when they received their first MB session, with an average gestational age of 29 weeks (range 10–39). Eighty-eight percent of clients received MB 1-on-1 in English and 12% in Spanish.

### Home visitor feasibility and acceptability data

When asked to rate their ability to cover material in a session, home visitors indicated they completely covered 87.9% of the sessions they delivered. Sessions in the Overview module were totally covered most often (92.3%), while the sessions in the other three modules were each totally covered about 85% of the time. Home visitors indicated that clients totally understood MB material 82.5% of the time when averaged across all sessions. There were, however, discrepancies in the extent to which home visitors felt clients understood content in the different modules. Home visitors indicated that clients fully understood the material in the Pleasant Activities module 95.0% of the time, whereas home visitors felt clients totally understood the Contact with Others, Overview and Thoughts Modules less often (Table 1). There was also variability in the extent to which home visitors felt clients were engaged in MB sessions. Similar to home visitors’ ratings of client comprehension of MB materials, home visitors felt clients were most often totally engaged with the Pleasant Activities module (79.4%). Each of the other modules was rated as having clients totally engaged between 63.0–69.2% of the time. When asked via open-ended question to indicate challenges with implementing MB, the most commonly reported challenge was the presence of children who were crying or distracting the client from focusing, followed by preoccupation with other household issues. The most commonly reported challenge specific to the MB curriculum was clients not always completing personal projects between sessions. In addition, several home visitors noted that material in the Thoughts module was repetitious. Home visitors also commented that while the script in the Instructor Manual was useful, it felt too long at times and did not clearly indicate when the home visitor should engage in conversation with the client (Table 2).

**Table 1:** Client Acceptability Data and Home Visitor Acceptability and Feasibility Data

	Client Acceptability Data			Home Visitor Acceptability and Feasibility Data		
	% Rating Very Enjoyable (mean, range)	% Rating Totally Understood (mean, range)	% Rating Very Useful (mean, range)	% Rating Material Totally Covered	% Rating Client Totally Understood Material	% Rating Client as Totally Engaged
Across 15 Sessions	82.0	91.6	85.1	87.9	82.5	71.2
By Module						
MB Overview	81.8 (80.3–82.9)	89.8 (88.0–93.4)	86.1 (84.6–88.1)	92.3	78.7	69.2
Pleasant Activities	84.6 (81.3–87.5)	94.3 (92.5–96.0)	84.7 (80.6–88.0)	85.4	95.0	79.4
Thoughts	79.9 (75.0–81.6)	92.4 (88.1–95.5)	83.6 (80.0–86.8)	84.6	74.5	65.5
Contact with Others	84.4 (76.9–93.3)	92.9 (86.7–100.0)	86.5 (84.6–88.9)	85.7	80.1	63.0

Sessions 1–3 = MB Overview, Sessions 4–6 = Pleasant Activities Module; Sessions 7–11 = Thoughts Module, Sessions 12–15 = Contacts with Others module

**Table 2:** Challenges Related to Implementing MB: Illustrative Home Visitor Qualitative Responses

MB Implementation Challenge	Quote
Children Distracting Client	“Mother of baby was distracted by the baby’s crying. MOB had to try and quiet the baby.” “Mom was checking on the baby who was fussy.”
Client Preoccupied with other Household Items	“Mom busy with daily chores and is constantly distracted.” “Lots of people in the home today. Very noisy.”
Client Not Completing Personal Projects	“Client always says that she will try to do personal project but then will say she had too much going on to do it.” “Although the client did not complete the pleasant activity personal project, she did verbalize that she engaged in 3 pleasant activities.”
Repetition of MB Material	“Some parts of the materials are repetitive but I tried not to repeat too much.” “List of harmful thoughts was confusing—it had already been covered last week...”
Instructor Manual Needs to Better Facilitate Conversation	“The script is long. I wonder if open ended questions would engage the client better throughout the session.”

### Client acceptability data

Clients, generally speaking, found MB 1-on-1 sessions to be enjoyable with 82.0% indicating they found sessions very enjoyable when aggregating ratings across all 15 sessions. Pleasant Activities and Contact with Others modules were rated to be slightly more enjoyable than the MB Overview and Thoughts modules (Table 1). Across all MB sessions, clients said they totally understood 91.6% of the sessions. There were minor differences across modules, with clients indicating they understood the pleasant activities module slightly better than the other three modules (Table 1). When asked to indicate perceived usefulness of MB sessions, 85.1% of sessions were rated as very useful when analyzing across all sessions. There was little variation in ratings of usefulness across modules or individual sessions (Table 1). Open-ended comments from client acceptability surveys highlighted several areas that clients particularly enjoyed. In particular, several clients noted they enjoyed using the curriculum’s “Quick Mood Scale” as an approach to keep track of their mood during the week. Other clients indicated that they enjoyed developing a list of pleasant activities that they could do by themselves or with others, including their child(ren), and discussing helpful and harmful thoughts (Table 3). Clients also reported on several topics they found useful, including the discussion of how to manage stress (Session 1). Clients consistently highlighted the skills taught in the curriculum as being useful, including generating a list of pleasant activities and strategies to stop harmful thoughts (Table 3).

**Table 3:** Client Enjoyment and Usefulness of MB Intervention: Illustrative Qualitative Responses

Client Acceptability Domain	Quote
Enjoyment of MB Intervention	“I really enjoyed the mood scale. I liked to learn how to recognize my mood”. “I enjoyed making a list of pleasant activities to do” “I enjoyed talking about helpful and harmful thoughts. It’s good for me to pay attention to any harmful thoughts or helpful thoughts”.
Usefulness of MB Intervention	“I enjoyed the way it explains how to manage stress and how it actually describes my stressors and how to manage. Very helpful”. “It made me think about how to make a negative day better and what getting up and doing things can do for you”. “It was really helpful because I learned some strategies to stop harmful thoughts”.

## Discussion

Castro et al. (2004) note that one form of intervention adaptation pertains to modification of program delivery [37]. Accordingly, this study adapted an evidence-based postpartum depression prevention intervention—the Mothers and Babies Course—from a group to an individual modality. In general, clients found MB 1-on-1 to be enjoyable, easily understood, and useful. Home visitors reported excellent fidelity in delivering the intervention and, in general, felt that clients understood the material. However, there appeared to be some discrepancies between home visitor and client ratings of comprehension for specific MB modules. In particular, home visitors felt that clients were less likely to fully understand content in the Thoughts module. Open-ended comments by home visitors also indicated some concern with the repetition of the Thoughts module, as well as a lack of clarity regarding when to engage in conversation with clients instead of doing didactic presentation of material.

In response to these client and home visitor feasibility and acceptability data, we have further modified the MB 1-on-1 curriculum. First, we have shortened the curriculum from 15 to 12 sessions by eliminating two Thoughts sessions and consolidating the Overview sessions into two sessions. The Additional file 1 presents the revised 12-session MB curriculum. Second, we modified the Instructor’s Manual to indicate in each session where home visitors should engage clients in interactive activities, using call-out boxes within each session. Third, we have added details in the Instructor’s Manual on the importance of completing personal projects and strategies to facilitate their completion. The revised version of the MB 1-on-1 curriculum is now being more formally tested via a randomized controlled trial to determine the efficacy of the curriculum in improving maternal mental health outcomes.

Several limitations to this study should be noted. Despite survey instructions to provide honest feedback, some social desirability bias among clients and home visitors may have occurred. In particular, it is possible that home visitors may have overstated client engagement in intervention content in an attempt to portray their intervention delivery in a more favorable light. We were also limited in our measurement of certain constructs. For example, client comprehension of the MB curriculum could have been assessed by asking clients to recall key content after it was delivered to them by their home visitor. Similarly, home visitors’ coverage of session material could have been examined using audio and/or video recordings that were rated by members of the research team. We opted to rely on self-report of these constructs at this stage of the research, in large part due to fiscal constraints. We plan more rigorous assessment of client comprehension and observational coding of intervention fidelity in future research.

## Conclusions

Given the encouraging findings regarding the feasibility and acceptability of MB 1-on-1, we are currently conducting a randomized controlled trial (RCT) to determine the efficacy of the curriculum in reducing depressive symptoms and preventing new incidence of depressive episodes. In conducting this RCT, we are paying particular attention to potential differences between our MB group and 1-on-1 modalities on promotion of social support, given the potential value of group social support provided via the MB group modality. Given the ubiquity of cell phone users who use text messaging [38], we are also exploring the use of text messaging to reinforce MB skills and encourage completion of personal projects. By virtue of its ability for delivery within home visiting, MB 1-on-1 has the potential to help destigmatize engaging in mental health services and eliminate additional appointments to access mental health services. By using staff already salaried by HV programs to deliver the intervention, we believe MB 1-on-1 has considerable promise to improve maternal mental health on a large scale and in a sustainable manner without increased cost to HV programs.

## Additional file

Additional file 1: Detailed Description of MB 1-on-1 Session Content. This file provides a detailed descripton of the key topics found in the 12 sessions of the revised Mothers and Babies (MB) 1-on-1 curriculum. (DOCX 28 kb)

## Abbreviations

HV: home visiting
MB 1-on-1: Mothers and Babies 1-on-1 version
MB: Mothers and Babies
OECD: Illinois Governor’s Office for Early Childhood Development
RCT: randomized controlled trial
REDCap: Research Electronic Data Capture
