# Supplementary material for: Adaptation of an evidence-based postpartum depression intervention: feasibility and acceptability of mothers and babies 1-on-1
Source: BMC Pregnancy Childbirth. 2018 Apr 11;18:93. doi: 10.1186/s12884-018-1726-0 (PMC5896030; doi:10.1186/s12884-018-1726-0)
Supplement: Supplementary file 1 — Detailed Description of MB 1-on-1 Session Content. This file provides a detailed descripton of the key topics found in the 12 sessions of the revised Mothers and Babies (MB) 1-on-1 curriculum. (DOCX 28 kb) [file 12884_2018_1726_MOESM1_ESM.docx]

| **Supplemental Material: Detailed Description of Mothers and Babies 1-on-1 12-Session Content** | |
| --- | --- |
| **MB**  **Module** | **MB Session Content** |
| **Introduction** | **SESSION 1**  Stressors that can affect the mother-baby relationship   - Highlight that life stressors affect how we feel emotionally and physically - Discuss how specific stressors might affect: The mother’s emotional health and physical well-being; The mother-baby relationship; The Baby - Identify common life stressors in participants’ lives, including those following birth of their child   How the Mothers and Babies Course can help you   - Instill hope that there are skills to manage stress and that the Mothers and Babies Course will discuss helpful ways to manage stress. - Help participants understand that once they learn these skills and recognize the skills they have already developed, they can pass them on to their children.   Purpose and Overview of Mothers and Babies Course   - Introduce the Mothers and Babies Course - Discuss how by making changes in the way we behave, think and the support we receive from others we can manage stress and feel better.   Personal Project assignment: My Parents, My Teachers video |
|  | **SESSION 2**  Your mood and your personal reality   - Explain the concepts of inner and outer reality. - Help participants understand the connection between thoughts, behaviors, contacts with others, and mood.   Introduction to Quick Mood Scale   - Explain the Quick Mood Scale - Facilitator models completion of the Quick Mood Scale for client   Personal Project assignment: Completion of Quick Mood Scale |
| **Pleasant Activities** | **SESSION 3**  Quick Mood Scale review   - Review the Quick Mood Scale, discuss how client felt about completing the Quick Mood Scale, and what she learned from tracking her mood. - Reinforce the purpose of the Quick Mood Scale: to notice how she is feeling and notice what occurs that affects her mood, so she can begin to make small changes to improve her mood when she is feeling stressed/down.   Violet and Mary’s Days (“Cartoon” to introduce Pleasant Activities module)   - What you do affects how you think and feel about yourself, others, and the world. - You can choose to do things that make you feel better. - Doing pleasant activities can actually create energy. - Pleasant activities are part of our outer reality.   How does what we do affect how we feel?   - When people do pleasant activities they often feel happier, are more likely to have positive thoughts about their lives, and are more likely to have positive contacts with other people. - Doing pleasant activities helps to balance our lives, especially when we are feeling stressed. - Many mothers put the needs of everyone else in the family first, and their needs last   Personal project assignment: Brainstorm personal activities |
|  | **SESSION 4**  What do you like to do? Pleasant activities list   - Help participant identify activities she enjoys doing both alone and with her baby. - Some pleasant activities are brief, cost nothing, and can be a part of everyday routines, if we can take time to notice and enjoy them. - We don’t need to do a lot of pleasant activities to feel good. - Mothers can do activities with their babies that can affect both their mood and their babies’ mood - Pleasant activities are part of our outer reality. Doing pleasant activities affects how you feel and it changes both your outer reality (what you are doing) and inner reality (how you feel).   Overcoming obstacles to doing pleasant activities   - Help participants identify obstacles to doing pleasant activities. - Discuss ways they might overcome these obstacles. Discuss problem solving as one way to overcome a roadblock or problem.   Personal project assignment: Schedule a pleasant activity for upcoming week |
|  | **SESSION 5**  Review of personal project   - Discuss whether participants were able to complete the pleasant activity they scheduled. - Emphasize that it is sometimes difficult to complete pleasant activities even when they are scheduled, but it is important to not give up on scheduling and doing pleasant activities. - Discuss ways they might overcome obstacles, if any existed.   What do babies like to do?   - Ways to engage babies in pleasant activities - How do babies learn? - Doing pleasant activities with babies/infants   Personal project assignment: Completion of Quick Mood Scale, including keeping track of pleasant activities each day |
| **Thoughts** | **SESSION 6**  Violet and Mary’s Days (“Cartoon” to introduce Thought module)   - There are many different types of thoughts that one can have in any given situation. - These different thoughts can affect how we feel. - We have some control over what we think, and can better manage our inner reality.   What are thoughts?   - Thoughts are like self talk, like having a conversation with ourselves. - There is a relationship between our thoughts and our mood. Thoughts are part of our inner reality, and our inner reality is related to our mood/emotions. - Our thoughts can affect the way we feel, can affect our bodies (e.g. tension), and can affect what we do.   Helpful thoughts and harmful thoughts   - Helpful thoughts help improve mood. - Harmful thoughts worsen mood. - Both helpful and harmful thoughts affect us emotionally and physically and affect our inner reality. - It is important to understand how the different thoughts we have can affect our mood - Identifying harmful and helpful thoughts about your pregnancy and how they affect your mood is an important step toward improving your mood.   Personal project assignment: Keep track of helpful and harmful thoughts |
|  | **SESSION 7**  Types of harmful thought patterns and strategies to “talk back” these thoughts   - E.g. overgeneralization, negative fortune telling, all or nothing thinking, blaming oneself   Ways to change harmful thoughts that affect mother and baby   - There are a number of strategies for changing harmful thoughts. - Each strategy can be used both to reduce our harmful thoughts and to teach our children how to have a healthy mood. - E.g. thought interruption, worry time, time projection, self-instruction   Personal project assignment: Quick Mood Scale and use of two skills to reduce harmful thoughts |
|  | **SESSION 8**  Thoughts about being a mother   - Children learn patterns of thinking from their parents. - The way mothers think about their children and themselves affects how they behave with their children, and this affects the way their children think about themselves, their mother, and their relationship.   Goals for me and my baby’s future   - Mothers play an important role in shaping their babies’ thoughts and inner reality, which can have an impact on both the mother’s and the baby’s mood.   How do thoughts affect our behavior?  Personal project assignment: Things you want and don’t want for your child’s future |
| **Contact**  **with**  **Others** | **SESSION 9**  Violet and Mary’s Days (“Cartoon” to introduce Contact with Others)   - Note the importance of the reciprocal nature of interpersonal problems and depression. - Mary and Violet have different ways of managing their outer reality, which affect their mood.   Relationship between mood and contact with others   - Provide education on the reciprocal nature of interpersonal problems and depression. - Identify participant’s current support system. - Contact with others is part of our outer reality.   Breaking the cycle between negative mood and fewer positive contacts   - Help participant identify how to break the cycle between negative mood and fewer positive contacts (or more negative contacts) with others   Personal project assignment: Completion of Quick Mood Scale, including keeping track of positive and negative contacts with others |
|  | **SESSION 10**  The people in my life/identification of one’s social support system   - Recognize the importance of social support and its relationship to mood. - Humans by nature are social beings. - Participants can identify and evaluate their own social support system. - We can make choices about who we spend time with.   People in my life and the ways they support me   - Participants can identify and evaluate their own social support system. - There are different kinds of support.   Personal project assignment: Identification of people who can support your child |
|  | **SESSION 11**  Communication style and ways it affects your mood   - Identify participants’ primary style of communication (passive, assertive, aggressive) in interpersonal situations. - There are different communication styles that may work in different situations. - Communication styles can affect mood - Communication styles can affect relationships with others   Getting your needs met: use of assertive communication style   - Asking for help in a positive, clear, and direct way (being assertive) can increase the chance that one’s needs will be met (but not always). - One way to ask for help is to do it systematically (step by step approach). - By being assertive and expressing what you want and how you feel in a respectful way, you can improve relationships with others. - Identifying obstacles to being assertive or expressing one’s needs can help improve one’s outer reality. - There are different ways to overcome obstacles to expressing one’s needs.   Role changes and transitions and relationship to one’s mood   - A role change or transition—like becoming a new mother or having another baby—can affect your mood. - Sometimes even positive role changes can make you feel depressed because taking on a new role can be stressful. - Understanding how a role change is affecting you can help you feel less helpless and can improve your mood.   Personal project assignment: Make a request for support using assertive communication |
| **Wrap Up** | **SESSION 12**  Course review   - Review the main concepts: Relationships between mood and pleasant activities, thoughts, and contacts with others can affect one’s inner and outer reality. - Thoughts affect our inner reality. - The activities that we do and the people in our lives can affect our outer reality. - We can make choices to have a healthier reality (both inner and outer) and a healthier mood.   Planning for the future: How to continue to use skills learned in Mothers and Babies Course   - Encourage client to keep Mothers and Babies workbook for future reference. - Encourage client to continue using strategies that have been helpful and try out some of the skills she hasn’t tried yet. |
